# Correlation of Pneumonia Severity Index and CURB-65 Score with Neutrophil/Lymphocyte Ratio, Platelet/Lymphocyte Ratio, and Monocyte/Lymphocyte Ratio in Predicting In-Hospital Mortality for Community-Acquired Pneumonia: Observational Study

**DOI:** 10.3390/jcm14030728

**Published:** 2025-01-23

**Authors:** Aliye Gamze Calis, Burcu Karaboga, Fatih Uzer, Nermin Kaplan, Mustafa Karaca, Rojan Barış Gedik, Ahmet Alper Durmuş

**Affiliations:** 1 Chest Disease Clinic, Antalya Training and Research Hospital, 07100 Antalya, Turkey; 2 Chest Disease Clinic, Ataturk State Hospital, 07070 Antalya, Turkey; 3 Department of Chest Disease, Akdeniz University School of Medicine, 07070 Antalya, Turkey; md.fuzer@gmail.com (F.U.);; 4 Department of Internal Medicine, Akdeniz University School of Medicine, 07070 Antalya, Turkey

**Keywords:** community-acquired pneumonia, neutrophil-to-lymphocyte ratio, monocyte/lymphocyte ratio, platelet/lymphocyte ratio, scoring systems, prognosis prediction

## Abstract

**Background/Objectives**: Community-acquired pneumonia is a major cause of morbidity and mortality, and various scoring systems and laboratory assessments are available for predicting prognosis. The untapped potential of combining the neutrophil/lymphocyte ratio (NLR) with the monocyte/lymphocyte ratio (MLR) and platelet/lymphocyte ratio (PLR) and their correlation with the pneumonia severity index (PSI) and CURB-65 motivated our research. We thought that this would provide more robust data for predicting CAP prognosis. We aimed to assess hematologic parameters’ associations with the PSI, CURB-65, and qSOFA scores for predicting the prognosis of hospitalized CAP patients. DESIGN AND SETTING: This is a multicenter, observational study conducted in three hospitals in Türkiye, Antalya. **Methods**: A total of 343 patients hospitalized with CAP in three centers in Turkey, Antalya, between 1 January 2020 and 30 September 2023 were retrospectively enrolled. The demographic data, comorbidities, vital signs, radiological images, laboratory findings, and 30-day mortality results of the patients were recorded. CURB-65, PSI, and qSOFA scores were calculated. **Results**: This study included 163 females (47%) with an average age of 74 ± 11.8. Hospital mortality occurred in 51 patients. Non-survivor CAP cases had higher ages (*p* = 0.007), CURB-65 scores (*p* < 0.001), PSIs (*p* < 0.001), and qSOFA scores (*p* < 0.001) and a longer hospital stay (*p* = 0.001) and total antibiotic duration (*p* < 0.001). Additionally, the NLR (*p* = 0.009), MLR (*p* = 0.018), and PLR (*p* = 0.025) were higher in the non-survivor group. The CURB-65, PSI, and qSOFA scores demonstrated strong predictive capabilities for in-hospital mortality. In the ROC analysis conducted to predict in-hospital mortality, the area under the curve (AUC) for CURB-65, the PSI, and qSOFA was determined to be 0.83, 0.82, and 0.82, respectively. The NLR correlated positively with CURB-65, the PSI, and qSOFA; the PLR correlated with the PSI and qSOFA; and the MLR correlated with CURB-65. **Conclusions**: CURB-65 and PSI scores remain highly effective for predicting in-hospital mortality in CAP patients, as demonstrated by their superior AUC values. While the NLR, MLR, and PLR showed weak predictive performance compared to these scores, their correlations suggest their potential as adjunctive markers.

## 1. Introduction

Community-acquired pneumonia (CAP) remains a significant global public health concern, contributing substantially to morbidity and mortality rates worldwide. Pneumonia ranks as the eighth leading cause of death in the United States and stands as the primary cause of death due to infectious diseases among individuals aged 65 and older [1]. The assessment of pneumonia severity and prognosis is pivotal in guiding clinical decision-making and optimizing patient outcomes. Among the various clinical scoring systems employed for this purpose, the pneumonia severity index (PSI) and the CURB-65 score have emerged as widely utilized tools. These scoring systems incorporate clinical parameters to stratify patients based on disease severity, aiding in risk assessment and treatment planning.

Despite the established utility of PSI and CURB-65 scores, uncertainties persist regarding their precision and reliability in predicting critical outcomes such as in-hospital mortality and length of stay. Incorporating laboratory parameters into these scoring systems can make them more accessible and practical, particularly in settings with limited resources. Such integration could simplify decision-making and improve the efficiency of patient evaluations, especially in environments where time and resources are constrained. The integration of laboratory parameters into prognostic models has the potential to enhance predictive accuracy and refine risk stratification. This study endeavors to comprehensively explore and compare the efficacy of the PSI and CURB-65 score with laboratory parameters in forecasting in-hospital mortality among patients diagnosed with CAP. Nevertheless, physicians do not always utilize these commonly used scores associated with CAP in their everyday practice, mainly due to the extensive number of variables needed for their calculation [2,3].

In recent studies, the prognostic value of the neutrophil/lymphocyte ratio (NLR) along with the monocyte/lymphocyte ratio (MLR) and platelet/lymphocyte ratio (PLR) was investigated to predict mortality in bacterial and viral pneumonias by using the success of laboratory parameters in demonstrating the inflammatory process [4]. In another recent study, it was shown that the NLR, MLR, and PLR were significantly higher in a group of patients with pneumoconiosis that developed lung infection, indicating inflammation [5].

As patients’ leukocyte counts are monitored daily, the NLR, a simple and cost-effective marker of inflammation, has recently been proposed as a prognostic indicator for various infectious diseases, including pneumonia and periprosthetic joint infections [6,7,8,9]. However, the potential of combining the NLR with the MLR and PLR and their correlation with the PSI and CURB-65 has not been evaluated. By scrutinizing the interplay between clinical scoring systems and objective laboratory data, we aim to contribute valuable insights into the nuanced dynamics of pneumonia prognosis in patients diagnosed with severe pneumonia and admitted to an inpatient facility. The outcomes of this investigation may not only inform clinicians in their decision-making processes but also pave the way for a more nuanced and individualized approach to the management of pneumonia, thereby improving overall patient care and outcomes.

## 2. Materials and Methods

### 2.1. Study Design and Population

This study is a multicenter, retrospective, observational study. Patients admitted to chest disease clinics with a diagnosis of CAP in three centers in Türkiye, Antalya, between 1 January 2020 and 30 September 2023, were included in this study. Eligible participants were aged 18 years or older and diagnosed with pneumonia. Exclusion criteria included the diagnoses of organized pneumonia, viral pneumonia, fungal pneumonia, interstitial pneumonia, or pulmonary edema and incomplete file information. Laboratory parameters measured on admission were recorded. Complete blood count (CBC) included white blood cells (WBCs), neutrophils, monocytes, lymphocytes, and platelets. We used these parameters to calculate the NLR, MLR, and PLR. Also, CRP, kidney function test, and serum electrolyte results were collected.

### 2.2. Pneumonia Case Definition [10,11]

To be classified as pneumonia cases, patients needed to meet the following criteria within the first 48 h of hospital admission: identification of pulmonary infiltrations on chest X-ray or computed tomography, presence of productive or dry cough, body temperature above 37.8 °C or hypothermia below 36 °C, and presence of at least one systemic inflammatory marker (leukocytosis > 10,000 mm^3^, leukopenia < 4000 mm^3^, elevated CRP or procalcitonin values) [10].

### 2.3. Severity Assessment Tools

CURB-65 Scoring

CURB-65 was applied according to the following criteria [12]:

C—Confusion—1 point.

U—Blood Urea Nitrogen (BUN) level greater than 7 mmol/L or greater than 20 mg/dL—1 point.

R—Respiratory rate exceeding 30 breaths per minute—1 point.

B—Systolic blood pressure less than 90 mmHg or diastolic blood pressure less than 60 mmHg—1 point.

65—Age greater than 65—1 point.

Patients with a CURB-65 score higher than 2 were considered to be at high risk for mortality.

Pneumonia severity index (PSI) [13]:

The PSI is a scoring system used to assess the severity of pneumonia in adults. It helps healthcare professionals make decisions about the appropriate level of care for individuals with CAP. The PSI takes into account various clinical and demographic factors to stratify patients into different risk classes. These risk classes help guide decisions about whether a patient can be treated as an outpatient, requires hospitalization, or needs intensive care. The factors considered in the PSI include age, coexisting medical conditions, vital signs, laboratory results, and other clinical findings. The PSI is divided into five risk classes, ranging from I to V, with Class I being the lowest risk and Class V being the highest risk. The higher the class, the greater the perceived severity of the pneumonia, and the more likely hospitalization or intensive care may be needed.

qSOFA [14]:

qSOFA is a simplified scoring system designed to quickly assess the severity of illness in patients with suspected infections, particularly those with sepsis. qSOFA is intended to be a rapid bedside tool for healthcare providers to identify patients at a higher risk of poor outcomes. qSOFA consists of three components, and one point is assigned for each of the following:

Altered Mental Status: A Glasgow Coma Scale (GCS) score less than or equal to 13.

Systolic Blood Pressure: A systolic blood pressure of 100 mm Hg or less.

Respiratory Rate: A respiratory rate of 22 breaths per minute or more.

Patients with a qSOFA score of 2 or higher are considered to be at an increased risk of mortality and adverse outcomes. qSOFA is not a definitive tool for diagnosing sepsis, but rather, it helps identify patients who may need more intensive monitoring and management.

### 2.4. Ethical Approval

The ethical approval for this study was granted by the Clinical Research Ethics Committee (Internal Review Board) of Akdeniz University Faculty of Medicine with decision number KAEK-294 on 5 April 2023. The Declaration of Helsinki was adhered to throughout this study.

### 2.5. Statistical Analysis

A statistical analysis of the data was conducted using the SPSS 23.0 (SPSS, IBM, Chicago, IL, USA) version 23 software program. Categorical variables (sex, comorbidities, ICU requirement, mortality, etc.) were described as frequencies and percentages, while continuous variables (age, length of stay, CRP, PSI, CURB-65, qSOFA, white blood cells, platelets, neutrophils, lymphocytes, monocytes, NLR, MLR, PLR, etc.) were described as means and standard deviations. The normality of the data distribution was assessed using the Kolmogorov–Smirnov test. For normally distributed data, the means of two groups were compared using Student’s *t*-test, and the means of more than two groups were compared using a one-way ANOVA. For non-normally distributed data, the medians of two groups were compared using the Mann–Whitney U test, and the significance of categorical variables was analyzed using the chi-square test. The correlation between continuous variables was evaluated using the Spearman correlation test. To assess the prognostic ability of the PSI and CURB-65 score to predict mortality, both scores were calculated for each patient, and receiver operating characteristic (ROC) curves were generated. ROC curve analysis was performed, and values for area under the ROC curve (AUC), sensitivity, specificity, positive predictive value, and negative predictive value were calculated. Logistic regression analysis was performed to examine the effects of variables on mortality. Variables that were statistically significant in the binary comparisons were included in the logistic regression model. A significance level of 0.05 was considered in this study for statistical significance.

## 3. Results

**Figure 1:**
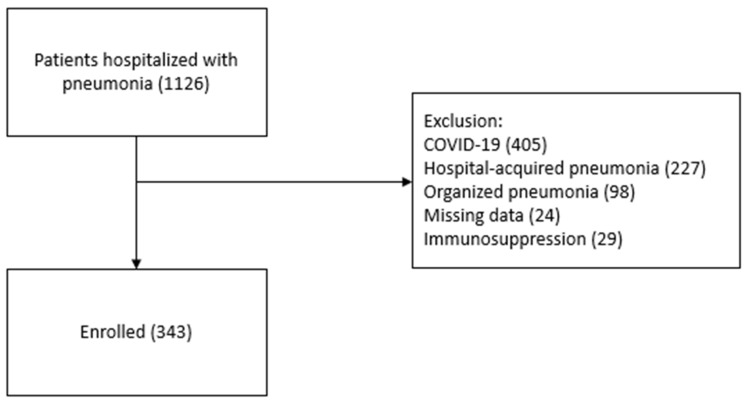
Flow chart of this study.

A total of 4985 patients hospitalized in chest disease clinics were retrospectively screened. Among these, 1126 patients hospitalized with pneumonia were identified. Of these, 405 were excluded due to the diagnosis of COVID-19 pneumonia, 227 due to hospital-acquired pneumonia, 98 due to the diagnosis of organized pneumonia, 29 due to immunosuppression, and 24 due to incomplete file information (Figure 1).

This study included 163 (47%) female and 180 (53%) male patients with an average age of 74.4 ± 11.8. Of the patients, 36.6% (127) had a history of smoking, and at least one comorbidity was present in the majority. The most common comorbidities were hypertension (55.3%), chronic lung diseases (41.5%), and diabetes mellitus (40.9%). Multilobar infiltrations were observed in 46.7% (n = 162) of patients on chest X-rays, and 31.1% (n = 108) required intensive care. Hospital mortality was detected in 51 (14.9%) patients. In 22.8% (n = 79) of cases, the initial treatment was revised to a broader-spectrum antibiotic. The mean CURB-65 score, qSOFA value, and PSI value for hospitalized CAP patients were determined as 1.7 ± 1.0, 0.6 ± 0.8, and 108.4 ± 34.6, respectively. The general characteristics of the patients are provided in Table 1.

**Figure 2:**
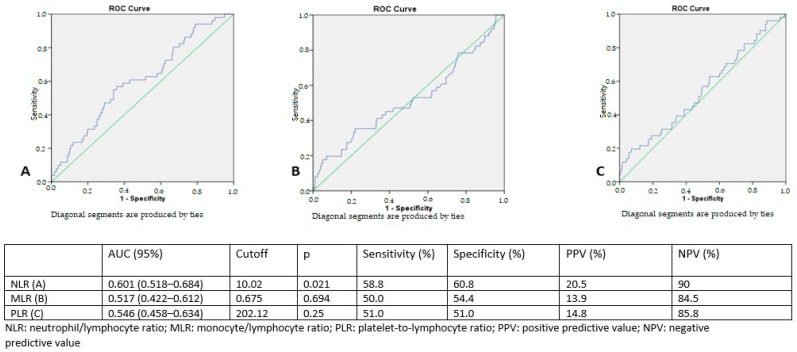
ROC curves for neutrophil/lymphocyte, monocyte/lymphocyte, and platelet-to-lymphocyte ratios predicting in-hospital morality.

The CURB-65, PSI, and qSOFA scores demonstrated strong predictive capabilities for in-hospital mortality. In the ROC analysis conducted to predict in-hospital mortality, the area under the curve for CURB-65, the PSI, and qSOFA was determined to be 0.83, 0.82, and 0.82, respectively. ROC curve analysis revealed that an NLR cutoff of 10.02 demonstrated a statistically significant association with in-hospital mortality, with a sensitivity of 58.8%, specificity of 60.8, positive predictive value of 20.5%, and negative predictive value of 90% (*p* = 0.021). The ROC curves for the NLR, MLR, and PLR are presented in Figure 2. 

When comparing the group of pneumonia patients who developed in-hospital mortality with those who survived, age (*p* = 0.007), CURB-65 score (*p* < 0.001), PSI (*p* < 0.001), qSOFA score (*p* < 0.001), length of hospital stay (*p* = 0.001), and total antibiotic duration (*p* < 0.001) were higher. In addition, the NLR (*p* = 0.009), MLR (*p* = 0.018), and PLR (*p* = 0.025) were higher in the mortality group. A comparison between the mortality and survival groups is presented in Table 2.

When examining the correlation of the NLR, MLR, and PLR with clinical scoring systems, it was found that the NLR was positively correlated with CURB-65, the PSI, and qSOFA; the PLR was correlated with the PSI and qSOFA; and the MLR was correlated with CURB-65 (Table 3).

Logistic regression analysis revealed that qSOFA scores (*p* = 0.005), the PSI (*p* = 0.040), and the MLR (*p* = 0.033) significantly influenced mortality (Table 4).

## 4. Discussion

In this study, we investigated the relationships of hematologic parameters with commonly used scores such as the PSI, CURB-65, and qSOFA to predict the prognosis of patients hospitalized with CAP; it was observed that the NLR exhibited positive correlations with all three scores, the MLR showed positive correlations with CURB-65, and the PLR demonstrated positive correlations with the PSI and qSOFA. Additionally, an NLR cutoff of 10.02 was found to indicate mortality with a sensitivity of 58.8% and specificity of 60.8%.

The NLR is a marker of inflammation that can be calculated from complete blood count values. Neutrophil count alone indicates inflammatory status; however, it may be insufficient to show disease progression as there may be “false negative” results. Lymphocyte count, on the other hand, reflects the immune status of an individual and generally decreases as the inflammatory disease progresses; however, this decreased response is seen at a late stage and is not sufficient to detect disease progression. However, the ratio of these two parameters is thought to be more reliable than neutrophil count or lymphocyte count alone in predicting patient survival.

The PSI and CURB-65 scores are known to predict the prognosis of CAP well. However, since they encompass numerous parameters, the search for a biomarker that could be more easily utilized in clinical practice continues. Some studies suggest that the NLR could be employed in predicting CAP. In the context of systemic inflammation, the demargination of neutrophils and the stimulation of stem cells via granulocyte colony-stimulating factor result in neutrophilia. Simultaneously, the accelerated apoptosis, margination, and redistribution of lymphocytes contribute to lymphocytopenia. In a prospective study involving 195 elderly patients with CAP, the NLR was assessed as a predictor of mortality, revealing an area under the curve of 0.95 [8]. In a study conducted by Huang and colleagues [15], it was observed that when the NLR was set at 2.2, it could distinguish patients with CAP from healthy individuals with 88.8% sensitivity. In our study, an NLR cutoff of 10.02 appeared to identify mortality with 58.8% sensitivity and 60% specificity. Furthermore, it was determined that the NLR is correlated with CURB-65, the PSI, and qSOFA, and it was found to be significantly higher in patients with fatal outcomes compared to those who survived. This finding indicated the potential utility of the NLR in predicting pneumonia prognosis.

The MLR is an emerging parameter proven to be a dependable factor in assessing the severity and prognosis of respiratory infections like tuberculosis. It is also linked to community-acquired pneumonia and systemic inflammatory response [15]. In a study conducted by Huang and colleagues [15], it was reported that the MLR could distinguish those with pneumonia from healthy individuals. In addition, there are studies indicating that monocyte levels in children reflect the severity and prognosis of CAP [16]. In our study, the MLR was found to correlate with CURB-65. Moreover, it was significantly higher in patients with in-hospital mortality compared to those who survived.

The PLR has shown associations with various pulmonary diseases. It is utilized in predicting the 90-day mortality of patients with acute exacerbations of COPD, aiding in the diagnosis of hypersensitivity pneumonia, and distinguishing patients with community-acquired pneumonia. Unlike other ratios, the PLR has been minimally utilized in investigating CAP prognosis. In a recent study by Enersen and colleagues [17], it was indicated that the PLR could be a valuable marker in indicating CAP prognosis. In our study, we observed higher PLRs in patients with fatal outcomes, and we found that the PLR is correlated with the PSI and qSOFA.

CURB-65 and the PSI are widely utilized tools that assess the mortality risk for patients with CAP, aiding clinicians in managing this condition. In our study, CURB-65 demonstrated an AUC of 0.83 for predicting in-hospital mortality, while the PSI had an AUC of 0.82 for the same prediction. However, the AUCs for the NLR, MLR, and PLR were 0.60, 0.51, and 0.54, respectively, indicating that they were not as strong in predicting mortality compared to the other parameters. The sensitivity and specificity of the NLR, MLR, and PLR, based on the cutoff values determined by their ROC curves, were also found to be low. Nevertheless, in our study, it was observed that CURB-65 exhibited positive correlations with the NLR and MLR, while the PSI showed positive correlations with the NLR and PLR.

The ROC curve results underscore the strong predictive capabilities of CURB-65 and the PSI, as reflected by their high AUC values of 0.83 and 0.82, respectively. These findings emphasize the utility of these scoring systems in accurately assessing mortality risk among hospitalized CAP patients, facilitating timely clinical decision-making and resource allocation. In practice, the ease of use and widespread familiarity of CURB-65 and the PSI make them invaluable tools in guiding treatment intensity and determining the need for intensive care. Moreover, their robust performance in this study reinforces their continued relevance as cornerstone methods for risk stratification in CAP management.

The present study had several limitations. At the outset, our focus was exclusively on hospitalized patients, resulting in a limited representation of individuals with minor illnesses, potentially introducing a bias to the findings. Another limitation of our study is that we only utilized parameters at the time of diagnosis and did not incorporate follow-up parameters. Additionally, in our study, there was no control group. Furthermore, serial measurements of the hematologic parameters were not performed, as this was beyond the scope of this study. Such serial assessments might have revealed potential temporal trends or differences, providing additional insights. Moreover, the exclusion of patients with suspected non-bacterial pneumonias (e.g., viral or fungal etiologies) based solely on clinical criteria represents another limitation, as the lack of detailed microbiological investigations may have introduced some diagnostic uncertainty.

## 5. Conclusions

In conclusion, the findings from this study highlight the continued clinical relevance of CURB-65 and PSI scores in predicting in-hospital mortality among patients with CAP. Both scoring systems demonstrated high AUC values of 0.83 and 0.82, respectively, reinforcing their effectiveness in risk stratification and guiding clinical decision-making. While this study identified associations between the NLR, MLR, and PLR with clinical scores like CURB-65, the PSI, and qSOFA, the predictive performance of these ratios in terms of sensitivity and specificity was lower compared to the established scores. Nevertheless, the positive correlations observed suggest their potential role as adjunctive markers in certain clinical settings. Future research should focus on validating these laboratory parameters in larger, diverse populations, as well as investigating their utility in follow-up assessments to enhance predictive accuracy.

## Figures and Tables

**Table 1:** The basic characteristics of the patients.

	N (%)
Female	163 (47.0)
Smoker (current/ex-smoker)	127 (36.6)
Hypertension	192 (55.3)
Diabetes mellitus	142 (40.9)
Chronic lung disease	144 (41.5)
Coronary artery disease	103 (29.7)
Cerebrovascular event	58 (16.7)
Malignancy	43 (12.4)
Multilobar	162 (46.7)
Intensive care unit admission	108 (31.1)
Monotherapy	171 (49.3)
Antibiotic upgrade	79 (22.8)
	mean ± sd
Age/yr	72.4 ± 11.8
CURB-65 score	1.7 ± 1.0
Length of stay/day	11.6 ± 10.8
qSOFA score	0.6 ± 0.8
PSI	108.4 ± 34.6
White blood cells (/mm^3^)	14,355.7 ± 7980.3
CRP (mg/L)	124.1 ± 110.5
HGB (g/dL)	11.6 ± 2.0
Platelets (/mm^3^)	266,249.7 ± 121,816.3
Neutrophils (/mm^3^)	11,848.0 ± 7471.8
Monocytes (/mm^3^)	936.9 ± 658.0
Lymphocytes (/mm^3^)	1430.8 ± 1115.7
Neutrophils/lymphocytes	13.1 ± 14.9
Monocytes/lymphocytes	0.8 ± 0.9
Platelets/lymphocytes	277.5 ± 254.7
Time of antibiotic upgrade/day	4.5 ± 2.2
Duration of antibiotic treatment/day	10.4 ± 7.2

**Table 2:** Comparison of survived and mortal patients.

	Patients That Died	Patients That Survived	*p*
Age/yr	76.5 ± 8.4	71.7 ± 12.3	0.007
Length of stay/day	16.2 ± 12.3	10.8 ± 10.4	0.001
CURB-65 score	2.9 ± 1.0	1.5 ± 0.9	<0.001
PSI	143.7 ± 30.2	102.3 ± 31.6	<0.001
qSOFA score	1.6 ± 0.8	0.5 ± 0.6	<0.001
CRP	148.6 ± 109.6	119.9 ± 110.3	0.088
White blood cells	14,548.8 ± 7364.7	14,322.5 ± 8092.9	0.852
Platelets	258,980.3 ± 140,636	267,502.1 ± 118,497	0.645
Neutrophils	12,484.5 ± 6860.3	11,738.4 ± 7577.6	0.511
Lymphocytes	1173.5 ± 713.0	1475.2 ± 1166.4	0.075
Monocytes	842.7 ± 517.5	953.2 ± 678.7	0.269
Neutrophils/lymphocytes	18.1 ± 22.1	12.2 ± 13.1	0.009
Monocytes/lymphocytes	1.2 ± 1.5	0.8 ± 0.8	0.018
Platelets/lymphocytes	707.3 ± 656.4	376.6 ± 375.1	0.025
Duration of antibiotic treatment/days	15.3 ± 11.6	9.6 ± 5.8	<0.001

**Table 3:** The correlation of the neutrophil/lymphocyte, monocyte/lymphocyte, and platelet/lymphocyte ratios with clinical scoring.

		CURB-65	PSI	qSOFA	NLR	MLR	PLR
CURB-65	r	1	0.659	0.591	0.175	0.115	0.181
	*p*		<0.001	<0.001	0.001	0.032	0.152
PSI	r	0.659	1	0.606	0.192	0.081	0.267
	*p*	<0.001		<0.001	<0.001	0.132	0.033
qSOFA	r	0.591	0.606	1	0.146	0.063	0.294
	*p*	<0.001	<0.001		0.007	0.239	0.018

PSI: pneumonia severity index; NLR: neutrophil/lymphocyte ratio; MLR: monocyte/lymphocyte ratio; PLR: platelet/lymphocyte ratio.

**Table 4:** Logistic regression analysis of factors affecting mortality.

	S.E	*p*	Exp(B)
Age (y)	0.020	0.494	0.986
Length of stay (hospital)	0.027	0.565	0.984
PSI	0.008	0.040	1.016
CURB-65	0.258	0.079	1.575
qSOFA	0.298	0.005	2.298
Duration of antibiotics (day)	0.037	0.147	1.055
NLR	0.022	0.163	0.970
MLR	0.219	0.033	1.0593
PLR	0.001	0.277	1.001

PSI: pneumonia severity index; NLR: neutrophil/lymphocyte ratio; MLR: monocyte/lymphocyte ratio; PLR: platelet/lymphocyte ratio.

## Data Availability

The data presented in this study are available on request from the corresponding author.
